# Retraction Announcement

**Published:** 2017

The following manuscript has been retracted from our November – December, 2016 issue. It has been retracted on a request from the authors as they forgot to properly acknowledge the source of their data. The authors are grateful to those who pointed out this mistake. - ***Editor***

***Retraction in:*** Pak J Med Sci 2016;32(6):1500-1505. doi: https://doi.org/10.12669/pjms.326.11460

***Link:*** http://pjms.com.pk/index.php/pjms/article/view/11460/4800

Effective role of lady health workers in immunization of children in Pakistan

Saira Afzal^1^, Azka Naeem^2^, Unaiza Shahid^3^, Wajiha Noor Syed^4^, Urva Khan^5^, Nayyar Misal Zaidi^6^

Retracted on February 7, 2017

